# Balancing Community and Hospital Care: A Case Study of Reforming Mental Health Services in Georgia

**DOI:** 10.1371/journal.pmed.1001366

**Published:** 2013-01-08

**Authors:** Nino Makhashvili, Robert van Voren

**Affiliations:** 1 Global Initiative on Psychiatry, Tbilisi, Georgia; 2 Mental Health Resource Center, Ilia State University, Tbilisi, Georgia; 3 Vytautas Magnus University, Kaunas, Lithuania

## Abstract

As one article in an ongoing series on Global Mental Health Practice, Robert van Voren and Nino Makhashvili provide a case study from Georgia on mental health care reforms.

*This case study is part of the* PLOS Medicine *series on Global Mental Health Practice.*

## Introduction

Psychiatric services in the former Soviet Union were characterized by high rates of institutionalization and a strong focus on biological treatment. In the post-Soviet states, these features remain—there is strong resistance to the introduction of modern, community-based, and user-oriented services [1]. In many cases, psychiatric reform programs have come to a halt or even been reversed [2]. It is against this backdrop that Georgia began a critical phase of its mental health reform program almost two years ago.

Georgia, which has a population of 4.4 million and ranks 75th on the United Nations Development Programme's Human Development Index, is one of the three Caucasian countries that regained independence in 1991. Its recent history has been turbulent. The country was ravaged by a bitter civil war from 1991 to 1993, the economy almost came to a standstill, and the health care system collapsed. It took until the end of the 1990s for basic health care services to be reestablished. Progress continued during the first years of this century, with health systems reforms that included moving away from the “Semashko system" (a Soviet system of state-owned health facilities and state-funded health professionals [3]), changes in health care financing and provision, development of private health care insurance, and the privatization of health care providers.

The recent *National Health Care Strategy 2011–2015* [4] developed by the Ministry of Labour, Health and Social Affairs (MoLHSA) stresses the importance of mental health care and of ensuring a balance between providing community-based and hospital-based mental health services. In this article, we provide an overview of the mental health reform process, including its complexities and challenges. The reform process is still very much in progress, which makes it difficult to assess its impact. Nevertheless, the case of Georgia might provide insights that can help other countries that are embarking on a similar mental health reform program.

## The Mental Health Situation

**Figure 1:**
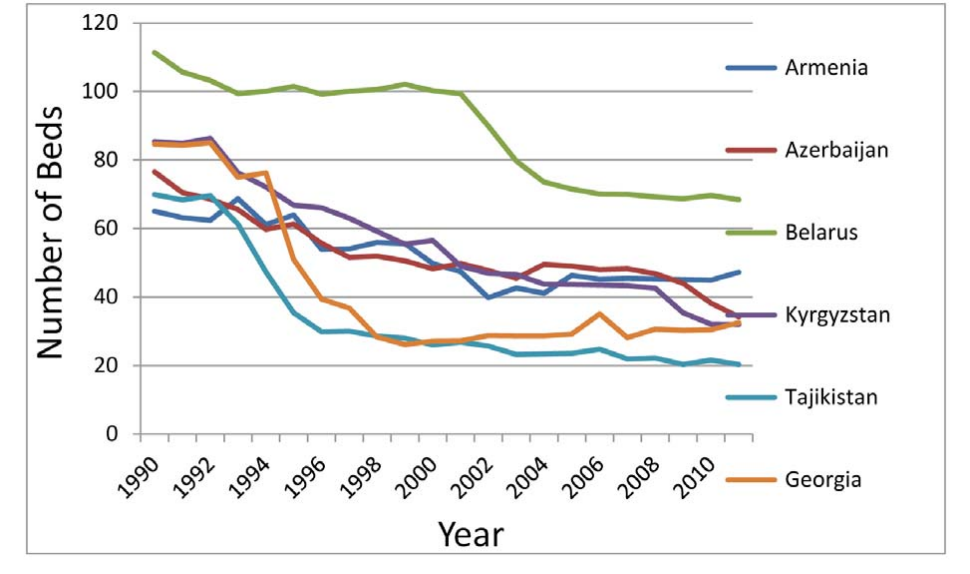
Beds in psychiatric hospitals (selected Commonwealth of Independent States countries).

In 1995, Georgia adopted a mental health care program (as part of a new general health care program) in which people with mental disorders on the national psychiatric register under the Ministry of Labour, Health and Social Affairs received free-of-charge services and treatment at both hospitals and outpatient clinics [5]. Six psychiatric institutions with an average of 1,000 beds provided hospital care (30.27 beds per 100,000 population). However, these mental health care reforms were accompanied by a significant decrease in funding for hospital beds, without providing any alternative outpatient care. This was a general trend in post-Soviet countries, as illustrated in Figure 1, which shows that there has been an almost five-fold reduction in the number of psychiatric beds since 1995, because of insufficient financing of mental health services [6]. Unfortunately, this decline in hospital services in Georgia was not counterbalanced by the development of outpatient and community-based services.

At present, aside from psychiatric hospitals, there are 18 outpatient psychiatric clinics (“dispensaries") in the country. However, there is an unequal distribution of mental health services across the country: there is less access, and a lower quality of services, in poor, remote regions. Nearly half (48%) of all licensed psychiatrists are working in the capital city, Tbilisi.

The number of people registered with a mental disorder in 2010 was 79,216 (out of a total population of 4.4 million) [7]. This figure is likely to be an underestimate of the true burden of mental illness, since it does not capture patients who visit private doctors or who do not access formal psychiatric services; thus, only those who have severe mental disorders are registered at dispensaries.

Health care expenditures have significantly increased over the past several years and in 2011 reached 10.1% of the country's gross domestic product [8]. However, only 2.11% of the total health budget is spent on mental health. Mental health care is delivered within the framework of the State Program for Mental Health Care and is administered by the MoLHSA. The budget of the program more than doubled between 2006 and 2011, reaching 12 million Georgian lari (US$7.3 million). Until recently, the state allocated about US$8–11 per day for patients admitted to institutions (2008–2010) and US$7–8 per day for outpatient treatment. This was hardly enough to cover salaries, heating, and food, which resulted in ineffective care. Table 1 shows changes in the state budget and services for psychiatric care between 2006 and 2011. The table illustrates a gradual increase in funding and diversification of the package of services that is offered to people with mental health disorders. However, it also shows the priority for funding of hospital care, the stagnation of funding for psychosocial rehabilitation, and the fact that only a very small portion of finances is reserved for outpatient care.

**Table 1:** Budget of Georgian state program for psychiatric care, 2006–2011 (in Georgian lari).

Service Components/Years	Year					
	2006	2007	2008	2009	2010	2011
Outpatient services	1,200,000 (24.2%)	2,000,000 (28.7%)	2,397,442 (28.7%)	2,597,232 (26.65%)	2,597,232 (26.1%)	2,833,600 (26.3%)
Psychosocial rehabilitation		50,000 (0.72%)	70,100 (0.84%)	70,100 (0.72%)	70,100 (0.7%)	70,100 (0.65%)
Hospital care	3,750,000 (75.8%)	4,900,000 (70.5%)	5,882,558 (70.5%)	6,933,780 (71.1%)	6,933,780 (69.7%)	7,170,200 (66.58%)
Child day care				100,688 (1%)	151,032 (1.5%)	120,000 (1.4%)
Urgent care				45,000 (0.46%)	45,000 (0.45%)	45,000 (0.4%)
Child mental health care in a general hospital						151,000 (1.4%)
Hospital Care of Substance Abuse conditions						144 000 (1.34%)
Crisis intervention and mobile service						236,100 (2.19%)
**Total**	**4,950,000**	**6,950,000**	**8,350,100**	**9,794,800**	**9,941,144**	**10,770,000**

Percentages in parentheses indicate the percent of each year's budget.

## Social Exclusion and Human Rights

Until recently, patients with mental health problems were kept in large institutions, where people were forced to live in inhuman conditions or sometimes even left to die [1]. Georgia has yet to complete the fundamental transformation from the old Soviet mental health care structure into a humane system that meets basic human rights standards [9].

Recent studies carried out in Georgia show the magnitude of the problem and reveal a strong link between mental ill health, social exclusion, and poverty [10]. Reports from the Public Defender's Office [11], based on regular monitoring of closed psychiatric institutions, highlight gross violations of all basic rights of inpatients. Such violations range from inappropriate involuntary hospitalization (which is now forbidden by the new Law on Psychiatric Care, introduced in 2007) to violations of a patient's right to privacy, information, and rehabilitation. The European Committee for the Prevention of Torture has repeatedly criticized the Georgian government for the poor conditions in the country's mental institutions [12],[13]. But the tide is now changing: the evidence on human rights violations that was presented to policymakers over the years was a strong impetus for the mental health reform process.

## The Push for Change

### The Legal Framework

One of the prime outcomes of human rights lobbying was the adoption of the new Law on Psychiatric Care [14], which is generally considered to be progressive and rights-based [15]. The law entered into force in 2007 and instituted a number of new practices, such as making a court decision for any involuntary hospitalization obligatory. Several bylaws introduced practical procedures, for example, procedures related to the use of physical restraint. In 2009, Georgian psychiatric care experts analyzed the law's implementation [16], and several further modifications were adopted, particularly related to procedures in forensic psychiatric treatment and prison mental health.

### The Crucial Involvement of Non-Governmental Organizations

One of the essential elements in the reform process was the strong voice of the non-governmental sector. The activity of civil society organizations, professional societies, user groups, and family member organizations created the momentum that was essential for a movement towards rights-based and humane mental health care. As representatives of one of these organizations, the Global Initiative on Psychiatry, we have intimate knowledge of the influence of the non-governmental organization (NGO) sector upon the mental health reform process. The NGO sector often functioned as the conduit for international expertise and knowledge about best practices in other countries. To provide an overview of NGO-originated interventions, we describe them here from the grassroots to the national level.

#### Reforms at the grassroots level

In searching for innovative, locally appropriate, and implementable models, new projects and activities were developed by mental health NGOs such as Global Initiative on Psychiatry and the Georgian Association for Mental Health, following World Health Organization [17],[18] and other international [19]–[21] recommendations. State standards regarding these new initiatives were adopted (e.g., regarding psychosocial rehabilitation and child day care service), and after they were proven to be effective and appropriate, these initiatives were replicated and integrated into the existing state health care system. Many new community-based services, such as crisis intervention and home care, were rolled out through this approach of small pilot projects followed by national scale-up. A recent example is the creation of crisis intervention teams that deal with emergency cases within certain catchment areas in the capital [22].

#### Reforms to mental health training

In challenging the old model of psychiatry and introducing contemporary approaches, capacity building activities have been promoted. These include the translation and publication of modern mental health literature into Georgian; the opening of the Mental Health Resource Center at Ilia State University in Tbilisi; a wide range of intensive trainings, workshops, and conferences; and the organization of exchange visits and research activities.

#### Reforms at the national level

At the national level, the main strategy of the NGO community was to influence the government and other mental health policymakers to adopt legislation and to abide by the new laws, to develop relevant mental health policies and plans (e.g., juvenile delinquency prevention), and to create monitoring mechanisms to ensure the protection of human rights. The efforts have been directed towards development of a coherent national mental health system. Some of the initiatives were successful, though they required long-term advocacy and much effort; others failed, such as the attempt in 2009 to introduce a mental health policy that would outline the direction reforms should take.

### International Donors

Many of the initiatives were made possible with funding from the international donor community. Whereas for many years the donor community often forgot to push for sustainability and embedding of programs within the local context, this changed after 2005. In the mental health field, the Dutch Ministry of Foreign Affairs, the European Commission, the United Nations Development Programme, and the Romanian Ministry of Foreign Affairs (channeling their funds through the United Nations Development Programme) provided the essential financial means to carry out pilot programs and finance them until local resources could take over.

## Reforms Take Shape

Several stages can be discerned in the process of reforming Georgian mental health care services. Increased funding as a result of the doubling of the state budget for mental health since 2004 allowed the MoLHSA to gradually scale up existing mental health services. This included improving the quality of treatment, rehabilitating some of the main psychiatric institutions, improving the living conditions of patients undergoing forensic treatment, and initiating a psychosocial rehabilitation program. In 2008 the introduction of a new funding model for hospital care gradually led to a reduction of the number of inpatients. However, these reforms still did not go far enough. Essential treatment methods, such as psychological treatment, remained unavailable, and there was still a lack of community services. Multidisciplinary teamwork and case management were still absent, and there was widespread low motivation, apathy, and resistance of the system to innovations. The long preparatory stage equipped the stakeholders with relevant knowledge and experience, which proved useful when designing further reforms. Acknowledging that “conditions, in which the patients of mental health care institutions live and undergo treatment, require urgent intervention," the MoLHSA announced a new and fundamental reform program at the end of 2010, and implementation started soon after.

The priorities of this recent program [23] are very much in line with international requirements and standards set by, for example, the World Health Organization [24],[25]. The MoLHSA's *National Health Care Strategy 2011–2015* [4] reiterated the importance of mental health care. The stated goal of this strategy is to improve the population's health by reducing disease burden and mortality by 2015. Strategic objectives include reducing inequalities in access to care; improving quality of services; protecting patients' rights; promoting prevention, preparedness, and response; and improving management of the health sector. A special chapter identifies “increased physical and geographical access to services" as a top priority and stresses the need to develop balanced, integrated, and continuous care for persons with mental disorders. To implement the desired changes, the MoLHSA created a Consultative Council on Reform consisting mostly of psychiatrists. High officials from the ministry take active part in the discussions and consultations.

## Initial Steps in the New Reform Process

### Deinstitutionalization

The most important dimension of the new reform process, deinstitutionalization, took place in early summer of 2011. Symbolically, the most significant step was probably the closing of one of the largest psychiatric hospitals in the country, the vast and dilapidated Asatiani Psychiatric Hospital in the center of Tbilisi, which had 250 beds at the time of its closure. Acute beds (in units of 30 beds) were relocated to newly opened psychiatric units in general hospitals (four departments are now functioning in multi-profile hospitals); a new child mental health ward with ten beds was opened in a general hospital; and a separate mental health center was established in Tbilisi, with a variety of services: an acute ward, a long-term treatment department, and an outpatient service, including a crisis intervention center with a mobile team. In addition, long-term residential facilities were opened in several towns (each with 40 beds), and crisis teams started functioning in some other cities of Georgia, for example, Batumi, Rustavi, and Kutaisi. Guidelines and codes of conduct were elaborated, and a service development policy was drafted.

These reforms immediately resulted in a fall in the length of stay for patients with acute mental illness, from an average of two to three months before the reforms to an average of 21 days now. The length of stay for a patient with acute mental illness refers to the time from initial hospitalization to either discharge or transfer to a long-term department.

For the next stage of the reform program, the MoLHSA plans to develop multifunctional community centers in three cities.

### Capacity Building

One of the priorities of the new reform program is the professional development of the mental health workforce. In 2011 a strategy for human resources development was elaborated, and basic modules for retraining were developed. Training for local professionals was led by European experts, and the first phase of retraining started in the summer of 2011. All mental health professionals from Tbilisi were invited to attend selected training courses and were enrolled free of charge. Pre- and post-course tests showed that 67% of the trainees acquired the necessary knowledge and skills. By now, more than 300 mental health workers have been trained; the basic training lasts 160 hours, and extended training lasts up to 240 hours. Regular supervision of workers by the expert trainers is provided to some services, to ensure proper implementation of acquired skills in the daily routine.

As in other former Soviet republics, mental health professionals have virtually no contemporary mental health literature in their own language. Western psychiatric literature was inaccessible in the Soviet Union for many decades. Although publication programs in the past 20 years have helped fill the gap, most of this literature was published in Russian, which many Georgian mental health professionals cannot read. The new reforms in Georgia attempt to tackle this problem with a publication program that has resulted in new textbooks of psychiatry in Georgian, as well as the first Georgian language manual for psychiatric nursing [26]. A glossary of mental health terminology is under development in order to standardize the language used in publications.

In October 2011, multidisciplinary working groups, which included service users, initiated a revision of the Georgian national clinical treatment guidelines for schizophrenia and depression. These revised guidelines have now been submitted to the MoLHSA for approval. Research is being carried out by a group of Georgian psychiatric experts to identify the most relevant topics in child and adolescent mental health care.

## Conclusions, Challenges, and Perspectives

Structural reform of a national mental health care system requires a long-term commitment. Such reform is likely to face repeated obstacles and setbacks that need to be overcome. Below we discuss four key challenges.

### 1. Developing a Clear Mental Health Plan

The MoLHSA needs to prioritize and clearly plan ahead—for example, the plan must account for the different mental health needs of people living in urban versus rural areas. An action plan for the coming years should be developed, which would help to link all existing and proposed mental health service components into one coherent and consecutive chain of services. This plan should include concrete strategies and activities to overcome financial and geographic barriers to accessing care, the development of a chain of well-coordinated community-based services, the integration of mental health into primary care, and the integration into the general care mental health care program of several domains such as prison mental health, psychotrauma care, and juvenile delinquency. The World Health Organization argues that the development and implementation of such a plan could have “a significant impact on the mental health of the population concerned" [17].

### 2. Improving Research Capacity

A robust research and information system should be put in place that collects and synthesizes relevant mental health data. Evidence is needed to demonstrate that services are effective and to justify the introduction of innovative care (which is often met with strong resistance). Evidence is also crucial in helping to guide sound policy decisions and to steer the reform process in the right direction.

### 3. Integrating Existing Services and Developing Care for Vulnerable Groups

One of the big challenges in the reform process is to integrate fragmented programs and services and to close the treatment gap by developing services that are needed for effective and continuous care.

Two major barriers to overcoming this challenge are the lack of psychosocial rehabilitation services and insufficient empowerment of service users. Though service users' voices are increasingly being heard and incorporated into the decision-making process, support programs for users are still scarce. The integration of health and social services is an essential element of the new reform process, yet achieving such integration is a huge challenge. Integration calls for a careful and diplomatic approach, since it requires overcoming vested interests and anxieties about future professional roles and positions. Similarly, the mental health care service within the Georgian penitentiary system requires major reforms [27], and it is vital to develop an appropriate care model and integrate it into general civil mental health services.

Another group that needs to be targeted for care is the war-affected population. The available data indicate high levels of psychological trauma, anxiety, depression, and substance abuse among members of war-traumatized communities [28]. The reform process needs to ensure that appropriate services are available to this group.

### 4. Overcoming Stigma and Resistance to Reform

Among the main factors that contribute to the continuation of ineffective and inhuman mental health care in Georgia are the stigma and discrimination that are widespread in the media, in governmental policies, and in society at large. In order to reduce stereotyping and discrimination, and promote more positive societal attitudes towards people with mental health problems, a major anti-stigma campaign is needed.

Resistance from service providers themselves is a last, but very important, challenge to mental health care advancement in Georgia, as in many other countries in the region. In general, psychiatrists might act as a considerable obstacle to the goal of closing the treatment gap [29]. This obstacle is widespread throughout former Soviet Union countries, where anxiety about the future is a general feature, and reform is often automatically seen as a risk to one's livelihood.

## Abbreviations

MoLHSA: Ministry of Labour, Health and Social Affairs
NGO: non-governmental organization
